# Transplantation of *Xenopus laevis* Tissues to Determine the Ability of Motor Neurons to Acquire a Novel Target

**DOI:** 10.1371/journal.pone.0055541

**Published:** 2013-02-01

**Authors:** Karen L. Elliott, Douglas W. Houston, Bernd Fritzsch

**Affiliations:** Department of Biology, University of Iowa, Iowa City, Iowa, United States of America; Columbia University, United States of America

## Abstract

The evolutionary origin of novelties is a central problem in biology. At a cellular level this requires, for example, molecularly resolving how brainstem motor neurons change their innervation target from muscle fibers (branchial motor neurons) to neural crest-derived ganglia (visceral motor neurons) or ear-derived hair cells (inner ear and lateral line efferent neurons). Transplantation of various tissues into the path of motor neuron axons could determine the ability of any motor neuron to innervate a novel target. Several tissues that receive direct, indirect, or no motor innervation were transplanted into the path of different motor neuron populations in *Xenopus laevis* embryos. Ears, somites, hearts, and lungs were transplanted to the orbit, replacing the eye. Jaw and eye muscle were transplanted to the trunk, replacing a somite. Applications of lipophilic dyes and immunohistochemistry to reveal motor neuron axon terminals were used. The ear, but not somite-derived muscle, heart, or liver, received motor neuron axons via the oculomotor or trochlear nerves. Somite-derived muscle tissue was innervated, likely by the hypoglossal nerve, when replacing the ear. In contrast to our previous report on ear innervation by spinal motor neurons, none of the tissues (eye or jaw muscle) was innervated when transplanted to the trunk. Taken together, these results suggest that there is some plasticity inherent to motor innervation, but not every motor neuron can become an efferent to any target that normally receives motor input. The only tissue among our samples that can be innervated by all motor neurons tested is the ear. We suggest some possible, testable molecular suggestions for this apparent uniqueness.

## Introduction

Synaptic contacts likely originated in evolution as connections between sensory-motor neurons and muscle tissue in diploblastic animals, such as jellyfish, [1] forming a monosynaptic reflex network from sensory cell directly to effector cell. As animal cell types diversified, the sensory-motor interface evolved in complexity, forming muscle fibers, motor neurons, sensory neurons, and interneurons [2]. As tissues diversified in the course of evolution into the over 200 cell types recognized in metazoans, motor neurons acquired novel targets during this diversification process. As new targets evolved to provide novel synaptic contacts, motor neurons evolved into novel sub-categories [3], [4], [5]. Although all motor neurons share a common developmental transcription factor, Islet 1 [6], [7], they have developed unique LIM code molecular signatures for each population [8]. Vertebrate motor (efferent) neurons have evolved to form synapses on a variety of targets originating from developmentally different sources including mesoderm-derived muscle fibers, epithelial placode-derived neurosensory cells and neurons (i.e. inner ear hair cells and neurons), and neural crest-derived autonomic ganglia [9], [10], [11], [12]. Among all cranial motor neurons, motor neurons of the facial nerve are unique in that they innervate targets from three different developmental origins: branchial arch-derived muscle by branchial motor neurons, neural crest-derived ganglia by visceral motor neurons, and placodally-derived inner ear hair cells by inner ear efferents [4]. Although the efferents to the inner ear hair cells project in mammals along the vestibulocochlear nerve, it has been shown that efferent innervation of the inner ear is ontogenetically derived from the facial branchial motor neurons, as inner ear efferent neurons exit the cell cycle in the same area as the facial branchial motor neurons in early embryonic mice prior to segregation of the two neuron populations [11], [13], [14] and can project with the facial nerve in the absence of afferent neurons [15]. Only one other branchial motor nerve, the glossopharyngeal nerve also innervates more than one tissue type: muscle, ganglia, and in aquatic vertebrates such as *Xenopus laevis*, hair cells of the placodally-derived posterior lateral line [16]. Given the ability of some motor neurons to innervate targets from a variety of origins, the possibility exists that there is conservation of the core molecular machinery that allows for recognition of a particular target by multiple motor neuron types.

In contrast to the affinity for few populations of motor neurons to innervate a variety of targets, most tissue types themselves generally have only one source of motor input, even when other motor neuron types project to adjacent territories. Such is the case with the innervation of the trapezius muscle by the spinal accessory nerve rather than spinal motor neurons [17], [18], [19]. Further support for specificity between motor neurons and targets includes studies in some vertebrates showing that spinal motor neurons can reinnervate the correct muscles following nerve transection [20]. In addition, spinal motor neurons can find the correct muscle following anterior-posterior reversal of a few lumbar spinal cord segments, demonstrating innervation selectivity [20],[21]. However, there still must be some degree of plasticity in the system, as the oculomotor nerve has been shown to innervate the lateral rectus muscle, a normal target of the abducens nerve, in Duane's retraction syndrome where the abducens nerve is absent [22] or the abducens nerve expands its territory when the oculomotor nerve is absent [23]. Furthermore, motor neurons were able to reroute to innervate a novel target, as demonstrated by *Xenopus laevis* spinal motor neurons rerouting to innervate hair cells of an ear transplanted to the trunk to replace a somite [24]. This result indicates the presence of a common molecular denominator between some targets of motor neurons, in this case between the inner ear hair cells and somites, that allows for recognition by the same population of spinal motor neurons. It is likely that the ability of different motor neurons to form synaptic contacts with hair cells of the inner ear uses an evolutionary conserved molecular mechanism for target recognition and for maintenance of innervation. Whether this applies to other motor neuron populations or other tissues is not yet known. Thus, our goal is to determine the extent to which various nerves can gain affinity for a novel target.

In the present study, we expand upon the previous *X. laevis* transplantation study [24] by transplanting *X. laevis* ears, which are directly innervated by motor (efferent) neurons into the path of various cranial motor neurons: oculomotor, trochlear, trigeminal, and, abducens to test for further acceptance of motor neuron innervation by hair cells. In addition, other tissues that receive direct motor innervation (somite-derived muscle and branchial arch-derived eye muscle and jaw muscle) were transplanted into the path of various cranial motor neurons or into the path of spinal motor neurons, respectively. Finally, tissues that are not directly innervated by motor neurons (heart, liver) were transplanted into the path of oculomotor, trochlear, and abducens motor neurons. Transplantation of the ear to the orbit revealed that motor neurons destined to innervate the muscles for eye movement could innervate hair cells of the transplanted ear if placed in their trajectory; however, comparable transplantations of other tissues revealed no innervation in somite-derived muscle, hearts, and livers transplanted to the orbit. In addition, neither eye muscle nor jaw muscle transplanted to the trunk to replace a somite was supplied by axons from spinal motor neurons. However, when somite-derived muscle was transplanted to replace an ear, there was supply of axons from what appears to be the hypoglossal nerve. Together these data suggest that the ear differs from other motor neuron targets in that it can receive motor neuron axons from a variety of sources and thus has a build in ability for novel motor neuron innervation.

## Materials and Methods

### Ethics Statement

All animal protocols used in these studies were approved by the Institutional Animal Care and Use Committee at the University of Iowa.

### Animals

*Xenopus laevis* embryos were obtained through induced ovulation using an injection of human gonadotropin and fertilized with a sperm suspension in 1× Marc's Modified Ringer's Solution (MMR). Embryos were kept at 18°C in 90 mm Petri dishes containing 0.1× MMR (diluted from 1× MMR, see below) until they reached stage 46 [25].

### GFP mRNA injections

For synthesis of green fluorescent protein (GFP) mRNA, plasmid template (pβGFP/RN3P) was linearized using SfiI, purified and mRNA synthesized using T3 RNA polymerase from the mMessage mMachine kit (Ambion). Protocol was followed according to manufacturer's directions.

The jelly coat was removed using 2% cysteine in 0.1× MMR pH 7.8 (diluted from 1× MMR, see below) shortly after fertilization. Embryos used for injection of GFP were placed in a Ficoll solution (2% Ficoll 400, GE/Pharmacia, in 0.5× MMR) for 5 min. GFP mRNA was diluted with RNase-free water so that the final amount injected was 1 ng. Embryos were injected at the 2 to 4 cell stage using a calibrated glass needle controlled by a Pico-Injector (Harvard Apparatus, Holliston, MA). Injections were made into each cell, keeping the total amount of mRNA per embryo constant.

### Dextran amine injections

Dextran amine [Fluorescein, 3000MW; FDA [26]] was dissolved in water to make a 1% solution. The jelly coat was removed as described above. Embryos were placed in the Ficoll solution (2% Ficoll) for 5 min prior to injection. Embryos were injected at the one cell stage using a glass needle controlled by a Pico-Injector.

### Transplantations of ear, somite, eye muscle, jaw muscle, heart, and liver

All transplantations were performed in 1× MMR pH 7.6–7.8, diluted from 10× stock (1M NaCl, 18 mM KCl, 20 mM CaCl_2_, 10 mM MgCl_2_, 150 mM Hepes) at room temperature under a dissecting microscope. For ear transplantations, otic placodes from the right side of stage 24–26 embryos were removed using fine tungsten needles and transferred ipsilaterally to the orbit, replacing the eye. In addition, otic placodes were removed from stage 24–26 embryos previously injected with GFP mRNA to label donor tissue and transplanted to the orbit of a non-injected host embryo of the same stage to replace the eye. For somite transplantations, donor somite tissue from stage 24–25 embryos previously injected with GFP was removed using fine needles and transplanted to the orbit of a host stage 24–25 embryo to replace the eye. Alternatively, donor somite tissue from stage 24–25 embryos were placed in a Fluorescein dextran amine solution (10,000MW, 10%) for 5 min, rinsed and transplanted into host stage 25 embryos to replace the ear. For eye-to-muscle transplantations, donor eyes and surrounding tissue from stage 25 embryos previously injected with GFP were removed using fine needles and transplanted to the trunk of a host stage 25 embryo to replace approximately three to four somites (the size of the eye). For jaw muscle transplantations, donor jaw muscle from stage 45–46 donor embryos previously injected with dextran amine (1%) were removed and processed into small pieces. A small jaw muscle fragment was transplanted to the trunk of a host stage 25 embryo to replace a somite. For heart transplantations, donor heart tissue from stage 27 embryos, some having been previously injected with GFP mRNA, was removed using fine needles and transplanted to the orbit of a host stage 24–26 embryo to replace the eye. For liver transplantations, donor liver tissue from anesthetized (0.02% Benzocaine) [27] stage 42 embryos was removed and transplanted to stage 24–26 embryos to replace the eye.

**Figure 1:**
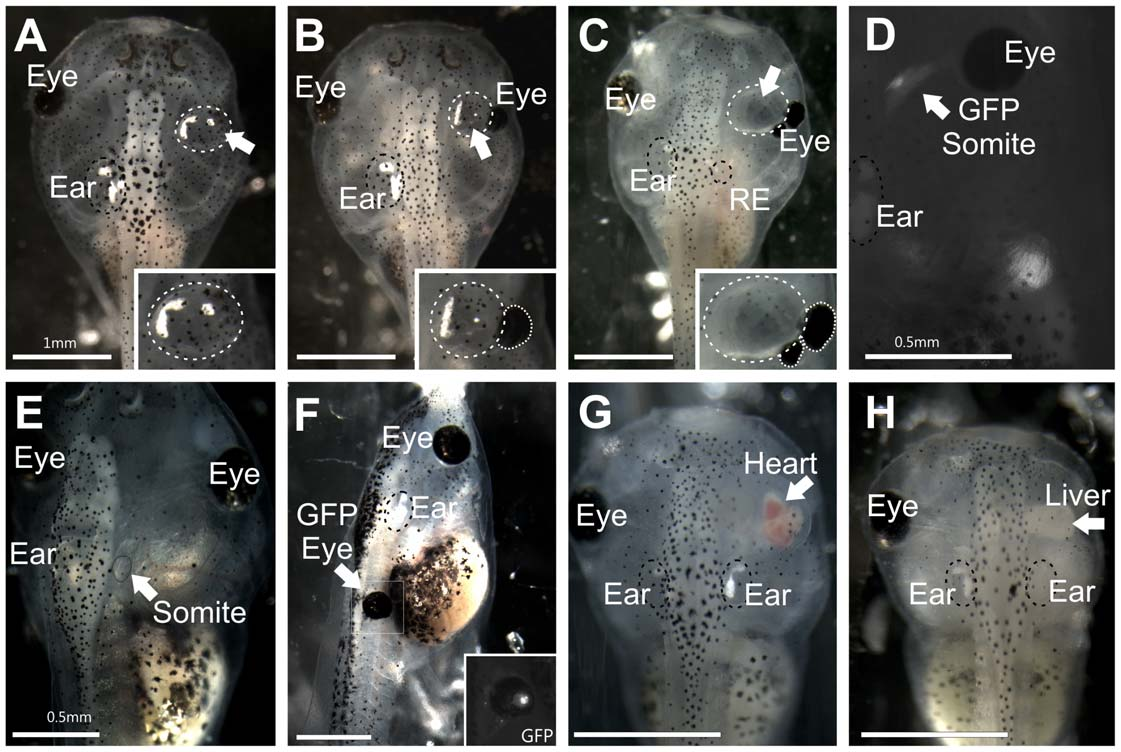
Stage 46 *Xenopus laevis*. **(A)** Embryo with a transplanted ear containing otoconia completely replacing the eye. Inset shows higher magnification of the transplanted ear. **(B)** Embryo with a transplanted ear containing otoconia medial to the reformed eye. A residual ear (RE) regrew in the native location. Inset shows higher magnification of the transplanted ear and remaining portion of the eye (circled). **(C)** Embryo with a transplanted, empty vesicle medial to the eye, which has formed a secondary eye more caudal. A residual ear regrew in the native location. Inset shows higher magnification of the transplanted ear and remaining portion of the eye (circled). **(D)** Embryo with transplanted GFP-expressing somite-derived muscle tissue medial to the eye. **(E)** Embryo with transplanted somite-derived muscle tissue to replace the ear. **(F)** Embryo with a transplanted donor GFP-expressing eye to the trunk, replacing a somite. Inset shows the GFP expression in the transplanted eye and surrounding transplanted eye muscle. **(G)** Embryo with a transplanted heart completely replacing the eye. **(H)** Embryo with a transplanted liver completely replacing the eye. Native, unmanipulated ears are labeled ‘Ear’ and are circled with a black dotted line. Eyes, native and reformed, are indicated by ‘Eye’. Arrows indicate transplanted tissues; transplanted ears are circled in addition with a white dotted line. Scale bar is 1 mm in A, B, C, F, H; 0.5 mm in D, E.

Embryos were kept in 1× MMR for 10–15 min post-transplant to promote healing before being transferred to 0.1× MMR. Healing was confirmed visually as a fusion of the ectoderm above the transplant. Transplants were monitored daily for continued growth. Completeness of transplanted ear formation was observed to determine the success of transplantations (Fig. 1A–1C). Success of GFP-positive ear transplantations was observed as the presence of a third ear (Fig. 1D) and was confirmed to contain GFP using an epifluorescence microscope. Success of other tissue transplantations was monitored (Fig. 1E–1H). Movies were recorded of beating hearts in the orbit using a camera mounted to a dissecting microscope (Leica). After embryos reached stage 46, they were anesthetized in 0.02% Benzocaine and fixed in 10% paraformaldehyde (PFA) by immersion.

### Lipophilic dye label

**Figure 2:**
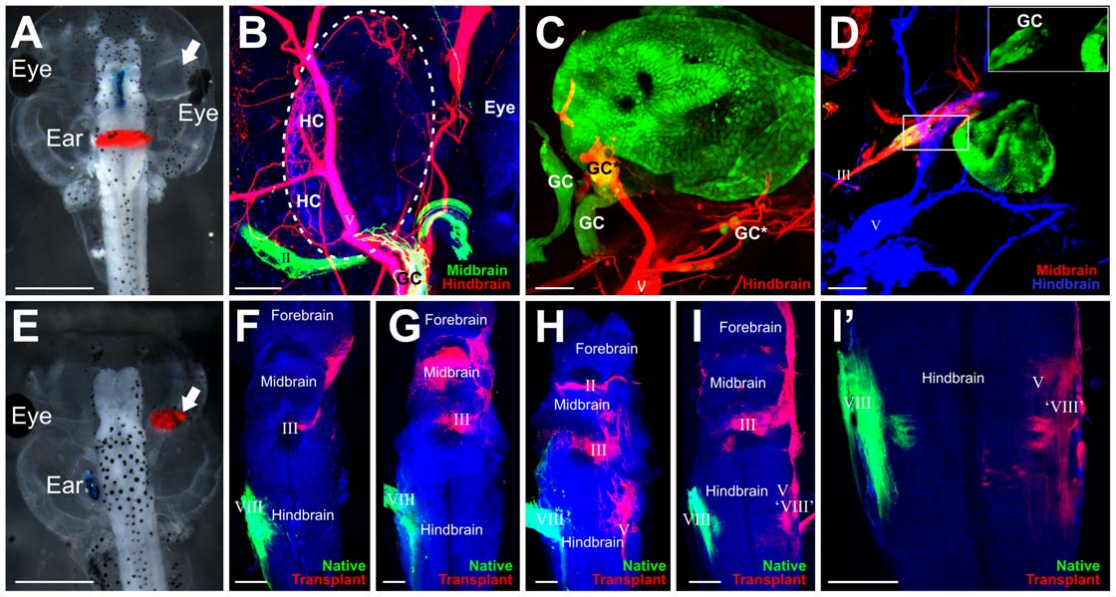
Afferent projections to transplanted ears. **(A)** Embryo showing implantation of lipophilic dyes into the midbrain (blue) and hindbrain (red). The transplanted ear is noted by the arrow. **(B)** Transplanted ear with ganglion cells (GC) projecting to hair cells (HC) in the inner ear and along the trigeminal nerve (V, red) back to the hindbrain. The optic nerve (II) is green. **(C)** Transplanted ear labeled with GFP reveals delaminated ganglion cells (GC), some of which project back to the brain (*) along the trigeminal nerve (V) as noted by colocalization with lipohilic dye. Other ganglion cells (**) did not colocalize with lipophilic dyes. **(D)** Transplanted ear labeled with GFP reveals delaminated ganglion cells (GC) which project back to the brain along the oculomotor nerve (III). Inset is higher magnification of boxed area showing the GFP labeled otic ganglion cells. **(E)** Embryo demonstrating implantations of lipophilic dyes into the native ear (blue) and transplanted ear (red, arrow). **(F–I)** Brains from embryos following lipophilic implantation into the native ear (green) and transplanted ear (red) reveal variation in afferent projections from the transplanted ear. Note: some of the lipophilic dye-labeled projections are from cranial nerves that were labeled transcellularly from the afferents. **(I′)** Stack of eight z-series confocal images from I showing hindbrain projections from the transplanted ear to the alar plate, probably the vestibular nucleus. Scale bar is 1 mm in A and E, 50 µm in B and C, 100 µm in D, F, G, H, I, and I′.

For tissues transplanted to the orbit, small pieces of dye-soaked filter paper [28], [29] were flattened and implanted longitudinally into the midbrain (NeuroVue™ Maroon) and transversely into the hindbrain (NeuroVue™ Red) at the level of the native ear (Fig. 2A), thus primarily filling the oculomotor nerve as it decussates the midline of the midbrain and the trigeminal nerve as it exits the hindbrain respectively. Both oculomotor and trigeminal nerves normally send projections to or near the orbit and thus near the transplanted tissue in the orbit. For some embryos, dye-soaked filter papers were implanted into the native and transplanted ears to label fibers projecting to the brain. Dyes were allowed to diffuse at room temperature for 24 hours, 36°C for 15 hours, or 60°C for 7 hours. Filter paper and brains were removed prior to imaging. Heads were mounted on a slide in glycerol and imaged with a Leica TCS SP5 confocal microscope.

For tissues transplanted to the trunk, small pieces of dye-soaked filter paper (NeuroVue™ Red) were flattened and implanted immediately ventral to the spinal cord. These were inserted from the contralateral side so not to destroy any nerves innervating the transplanted tissue. Dyes were allowed to diffuse at room temperature for 15 hours. Filter paper was removed prior to imaging. Trunks were mounted on a slide in glycerol and imaged with a Leica TCS SP5 confocal microscope.

For somite tissue transplanted to replace the ear, small pieces of dye-soaked filter paper were implanted transversely into the hindbrain at the level of the trigeminal nerve (NeuroVue™ Red) and at the level of the vagus nerve (NeuroVue™ Maroon). Filter paper was removed prior to imaging. Heads were mounted on a slide in glycerol and imaged with a Leica TCS SP5 confocal microscope.

### Immunohistochemistry

Heads containing the transplanted ears, somites, hearts, or liver were immunostained with antibodies against acetylated tubulin [30] or vesicular acetylcholine transporter (VAChT) [31] to label all nerves and motor neuron terminals, respectively, as described previously [24]. Concentration used for acetylated tubulin (Cell Signaling Technology) was 1∶800 and for VAChT (Sigma) was 1∶500. Species-specific secondary antibodies (Alexa) were used at 1∶500. Embryos were mounted on a slide in glycerol and imaged with a Leica TCS SP5 confocal microscope.

## Results

### Completion of eye removal and assessment of transplantation success

Completeness of transplanted tissue formation was scored to determine the effectiveness of transplantations (Figure 1A–1H; Table 1). Success for all tissues was defined as the detection of transplanted tissue, whereas completeness of transplantation was defined as the amount and/or degree of normality of transplanted tissue. Of the 159 successful ear to orbit transplantations (out of 173), almost two-thirds of the ears transplanted to the orbit contained otoconia (n = 97), whereas others were just a vesicle and lacked otoconia (n = 62) (Figure 1B–1C). Hair cells were present in ears that lacked otoconia (data not shown). Only ears containing otoconia were considered more complete ears and were used for further analysis. Removal of the lens placode and developing eye still lead to the regrowth of parts of the eye in all but 36 of the 159 successful ear to orbit transplantations (Compare inset in Fig. 1A with insets in Figs. 1B and 1C). Eye muscles, which develop from surrounding mesoderm, accompanied residual eyes. All embryos containing any remaining portion of the eye were used for further analysis.

**Table 1:** Success of tissue transplantation.

Transplantation	Transplanted Tissue Present	Transplanted Tissue Absent	Total
Ear to Orbit	159	14	173
Somite to Orbit	9	1	10
Eye/Eye muscle to Trunk	6	1	7
Jaw muscle to Trunk	12	1	13
Heart to Orbit	23	8	31
Liver to Orbit	13	0	13

Success of transplantation is defined as the detection of transplanted tissue.

Success of muscle tissue transplantation (somite, eye muscle, and jaw muscle) was also quantified by the presence of transplanted tissue (Figure 1D–1F). All but one transplant (out of 10) involving somites, whether to the orbit or otic region, were successful. Eyes with attached muscle transplanted to the trunk were present in 6 of 7 embryos. Attempts to transplant only eye muscle was not successful as no extra muscle was detected in the trunk when eye muscle was transplanted without the eye. Jaw muscle transplanted to the trunk was present in 12 of 13 embryos. Embryos with larger muscle transplants were used for further analysis. Success of heart and liver transplantations was also investigated (Figure 1G–1H). There was variation in the amount of heart tissue that developed in the orbit ranging from none detectable to a large, beating heart (Fig. 1G). Heart tissue was present in 23 of 31 transplants. Slightly less than half (10 of 23) of the hearts transplanted were beating regularly and apparently autonomously in the orbit. One transplanted heart beat at 130 beats per min (Fig. 1G), which was near the rate of native hearts (146 beats per min, n = 7). All 10 embryos containing beating transplanted hearts were used for further analysis. Liver tissue was present in the orbit in all 13 transplants and all were used for further analysis.

### Afferent innervation of transplanted ears

Lipophilic dyes implanted into the brain revealed sensory vestibular ganglion cells, detected by their cell bodies, labeled with dyes from both midbrain (blue) and hindbrain (red) implantations (Fig. 2A) when the ear was transplanted to the orbit. Most delaminating sensory ganglion cells sent projections along the nearby trigeminal nerve and into the hindbrain (Figs. 2B and 2C). Fewer ears sent projections along the oculomotor nerve and into the midbrain (Fig. 2D). Of the 25 transplanted ears analyzed with lipophilic dye tracing, 23 had lipophilic dye labeling of axons projecting to the transplanted ears. Twenty-one of these 23 ears had lipophilic dye labeling of sensory vestibular ganglion cells. Of these, 16 sent sensory axons along the trigeminal nerve only, 2 had ganglion cell axons projecting along the oculomotor nerve only, and 3 had projections along both trigeminal and oculomotor. However, not all ganglion cells may have sent projections to the hindbrain or midbrain as was evident in the transplanted ears labeled with GFP (Fig. 2C). In these ears (n = 4), some GFP-positive ganglion cells did not project to the brain areas where dye was implanted (Fig. 2C) as they showed no lipophilic dye label. It is not known where these unlabeled ganglion cells project to and if they reach the brain at all. Afferent axons that do reach the brain, as observed following implantations of lipophilic dye into the ear, project along either the trigeminal oculomotor or optic nerve to enter the brain; however, there was no consistency between different transplants in the trajectory of afferent projections (Fig. 2F–I). Of the eighteen ears in the orbit labeled with lipophilic dyes that sent afferents to the brain, one projected to the forebrain, midbrain, and hindbrain, five projected to the forebrain and midbrain, three to the midbrain alone, five to the midbrain and hindbrain, and four to the hindbrain alone. Afferent axons from three of the transplanted ears appeared to project to the ipsilateral vestibular nucleus (Fig. 2I, 2I′), a native target of the ear. These data suggest that projections of inner ear afferents primarily grow randomly along adjacent nerves with little evidence for preferences.

### Efferent innervation of transplanted ears

**Figure 3:**
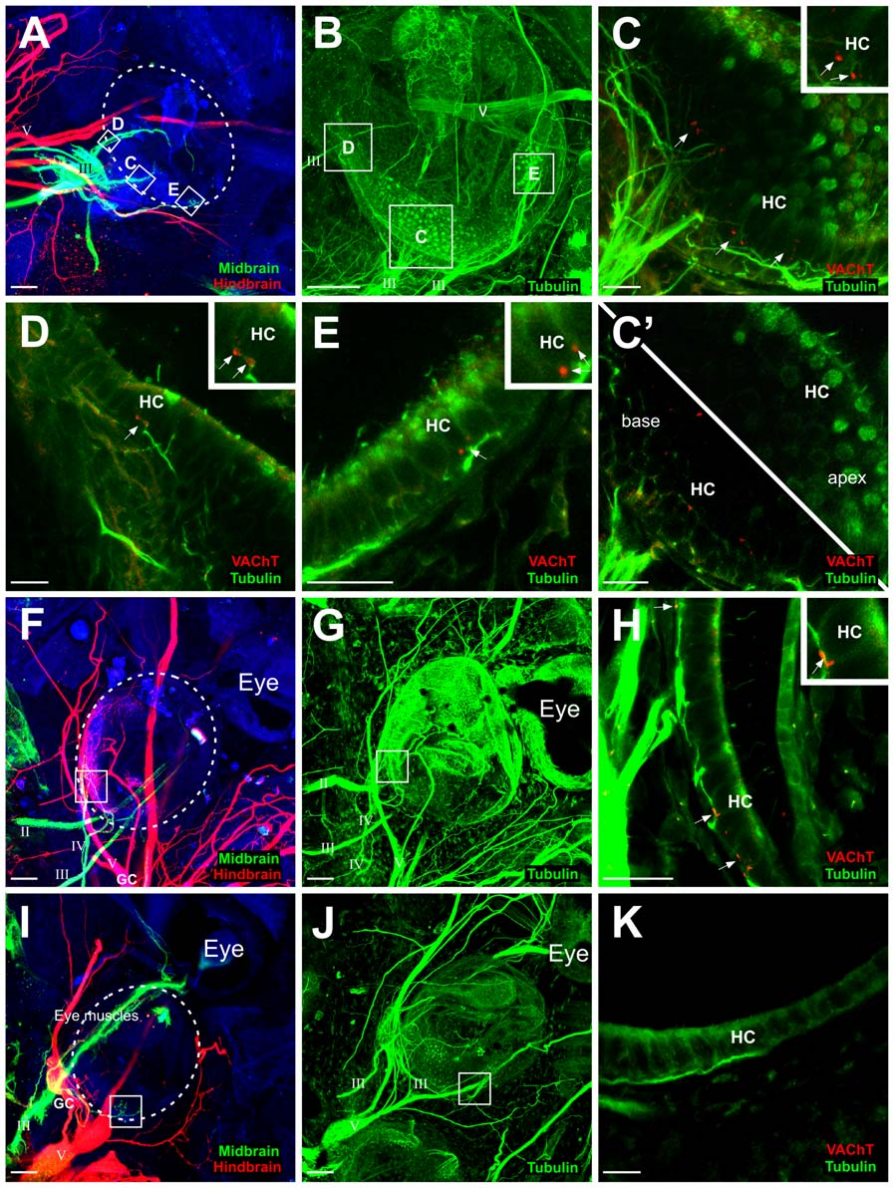
Efferent projections to transplanted ears. **(A)** Implantations of lipophilic dyes into the midbrain (green) and hindbrain (red) revealed axon projections from the oculomotor nerve (III) to hair cells of the transplanted ear (circled). **(B)** Immunohistochemistry for tubulin of the ear in A shows all innervation. **(C–E)** Immunohistochemistry for VAChT (red) confirms motor terminals on hair cells (HC) of boxed areas in B. Insets show higher magnification of VAChT staining at the base of hair cells. **(C′)** Single z-series images at the base of the hair cells (lower left) showing VAChT-positive terminals and at the apex (upper right) devoid of VAChT staining. **(F)** Implantations of lipophilic dyes into the midbrain (green) and hindbrain (red) revealed axon projections from the trochlear nerve (IV) to hair cells in the transplanted ear (circled). Afferent axons projected along the trigeminal nerve to the hindbrain, demonstrated by the colocalization of ganglion cells (GC) with the red lipophilic dye. **(G)** Immunohistochemistry for tubulin of the ear in G shows all innervation. **(H)** Immunohistochemistry for VAChT (red) confirms motor terminals on hair cells of boxed area in G. Inset shows higher magnification of VACHT staining at the base of hair cells. **(I)** Implantations of lipophilic dyes into the midbrain (green) and hindbrain (red) revealed ganglion cells (GC) projecting along the oculomotor nerve (III). For this ear, the oculomotor nerve innervated the eye muscles ventral to the transplanted ear. **(J)** Immunohistochemistry for tubulin of the ear in I shows all innervation. **(K)** Immunohistochemistry for VAChT (red) shows the absence of motor terminals on hair cells (HC) of boxed area in I. Scale bar is 100 µm in A, B, F, G, I, J; 25 µm in C, C′, D, E,H, K.

Efferent innervation of transplanted ears from the oculomotor and trochlear nerves was demonstrated with lipophilic dye implantation into the midbrain. Of the 25 embryos implanted with lipophilic dyes into their brains, 9 transplanted ears had projections to them from the oculomotor nerve (Fig. 3A) and 1 from the trochlear nerve (Fig. 3F). These projections were without obvious co-labeling of vestibular sensory ganglion cells, though a pure motor innervation required additional confirmation (see below). In 8 of 9 embryos in which the oculomotor nerve sent axons to the transplanted ear, the surrounding eye muscle was also innervated; the remaining embryo had no eye or eye muscles remaining to be innervated. In the embryo in which the trochlear nerve sent axons to the transplanted ear, eye muscles were present, but were not innervated. For the 13 embryos lacking oculomotor or trochlear nerve projections to the transplanted ear but had regrowth of part of the eye, the motor nerves innervated only the eye muscles. The most noticeable difference between embryos whose transplanted ears had projections to them and those whose did not was the position of the transplanted ear relative to the native eye muscle. All transplanted ears imaged that were more medial than the eye muscle received projections from motor nerves (5 of 5 ears). Half of the transplanted ears that were equidistant from the brain as the eye muscle received projections from motor nerves (5 of 10 ears). Finally none of the ears located lateral to the eye muscles were innervated (0 of 8 ears). These data imply that the ear is as good a substrate for motor neurons as are eye muscles with the decisive difference being driven by the relative position following a simple first encountered, first innervated rule.

It was necessary to confirm that there were indeed motor neuron axons projecting to the transplanted ear and not just afferent projections of sensory ganglion cells from the ear back to the midbrain along the oculomotor nerve, since ganglion cells were shown to occasionally project along the oculomotor nerve into the midbrain (Figs. 2D, 3I). Sensory ganglion cells were detected in these ears by the existence of neuronal cell bodies in a putative vestibular ganglion that were also labeled with lipophilic dye. Their axons could be traced back to the oculomotor nerve (Fig. 3I), or in other cases were indistinguishable from the nerve itself (Fig. 2D). We classified ears as receiving projections from oculomotor or trochlear nerve based on the absence of lipophilic labeling of sensory ganglion cells. These ear transplants without lipophilic dye-labeled neuronal cell bodies along the oculomotor or trochlear nerves were selected for further analysis. To further confirm that these axons projecting to the transplanted ear were of motor origin, we used an antibody against VAChT. Our previous data demonstrated that ears containing VAChT-positive axon terminals on hair cells observed with electron microscopy, had motor neurons with synaptic vesicles terminating at the base of hair cells [24], thus VAChT is a good indicator of motor innervation. We tested 12 ears that had lipophilic label when dye was inserted into the midbrain: 7 with projections from the oculomotor or trochlear nerve without labeled ganglion cells and 5 that had ganglion cells that were labeled (Fig. 2D) to serve as a control since these were not expected to have VAChT labeling. VAChT-positive motor axon terminals were confirmed on hair cells in the 7 transplanted ears determined to have motor neuron innervation based on lipophilic labeling (Figs. 3C–3E, 3H). The 5 ears that had ganglion cells labeled by midbrain lipophilic dye implantation (Figs. 2D, 3I) were not positive for VAChT (Fig. 3K). Immunohistochemistry for tubulin revealed innervation of the transplanted ears, demonstrating that axons from the oculomotor nerve was at least a subset of the total innervation of the ear, the remainder likely being afferents (Figs. 3B, 3G).

### Innervation of transplanted somite, eye muscle, and jaw muscle

**Figure 4:**
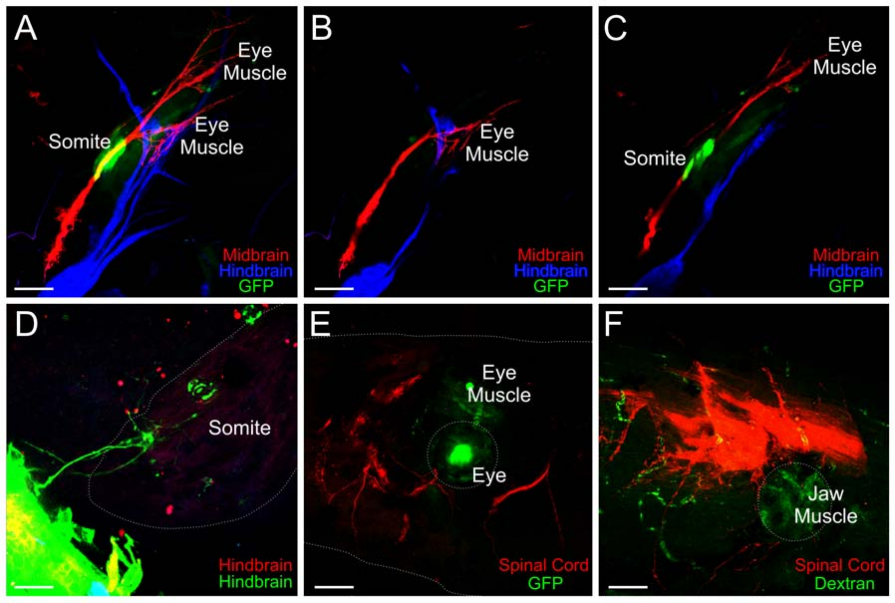
Transplanted muscle tissue. **(A)** Implantations of dye into the midbrain (red) and hindbrain (blue) revealed axon projections from oculomotor nerve to the eye muscles but not to the transplanted somite-derived muscle (GFP, green). **(B–C)** Single z series showing innervation to the eye muscle but not the transplanted somite-derived muscle (GFP, green). **(D)** Implantations of dye into the hindbrain at the level of the trigeminal (red) and the glossopharyngeal, vagus, and hypoglossal (green) revealed axons projecting to transplanted somite-derived muscle tissue, likely from the hypoglossal nerve. **(E)** Implantations of dye ventral to the spinal cord revealed spinal motor neuron innervation of surrounding somite-derived muscle but not to the GFP-positive eye muscle (green) transplanted with the eye. **(F)** Implantations of dye ventral to the spinal cord revealed spinal motor neuron innervation of surrounding somite-derived muscle, but not to the jaw muscle transplanted from a dextran-injected embryo. Scale bar is 100 µm.

Somite-derived muscle transplanted to the orbit failed to be innervated by oculomotor or trochlear nerves (0 of 14 transplants) as demonstrated by absence of innervation when lipophilic dyes were implanted into the midbrain. Even when the somite-derived muscle was located medial to the native eye muscles as was the case for 3 transplants, the oculomotor nerve bypassed the somite-derived muscle to innervate the remaining eye muscles (Figures 4A–4C). In contrast, when the somite-derived muscle was transplanted to the otic region to replace the ear, 3 of 5 transplants showed some projections of possibly motor neuron axons to the somite-derived muscle, likely by the hypoglossal nerve. This suggestion derives from implantations of lipophilic dyes implanted into the hindbrain rostral and caudal to the transplanted somite (Figure 4D). The remaining 2 transplants were not innervated.

Eye muscle transplanted with the eye to the trunk failed to be innervated by spinal motor nerves (0 of 4 transplants) as demonstrated when lipophilic dyes were implanted ventral to the spinal cord to label motor neurons as they exit the spinal cord (Figure 4E). In addition, jaw muscle transplanted to the trunk failed to be innervated by spinal motor nerves (0 of 9 transplants) as demonstrated when lipophilic dyes were implanted ventral to the spinal cord (Figure 4F). For both eye muscle and jaw muscle transplants, the spinal motor neuron axons exiting the spinal cord navigated around the transplanted tissue and innervated neighboring native somite-derived muscle.

### Innervation of transplanted heart and liver

**Figure 5:**
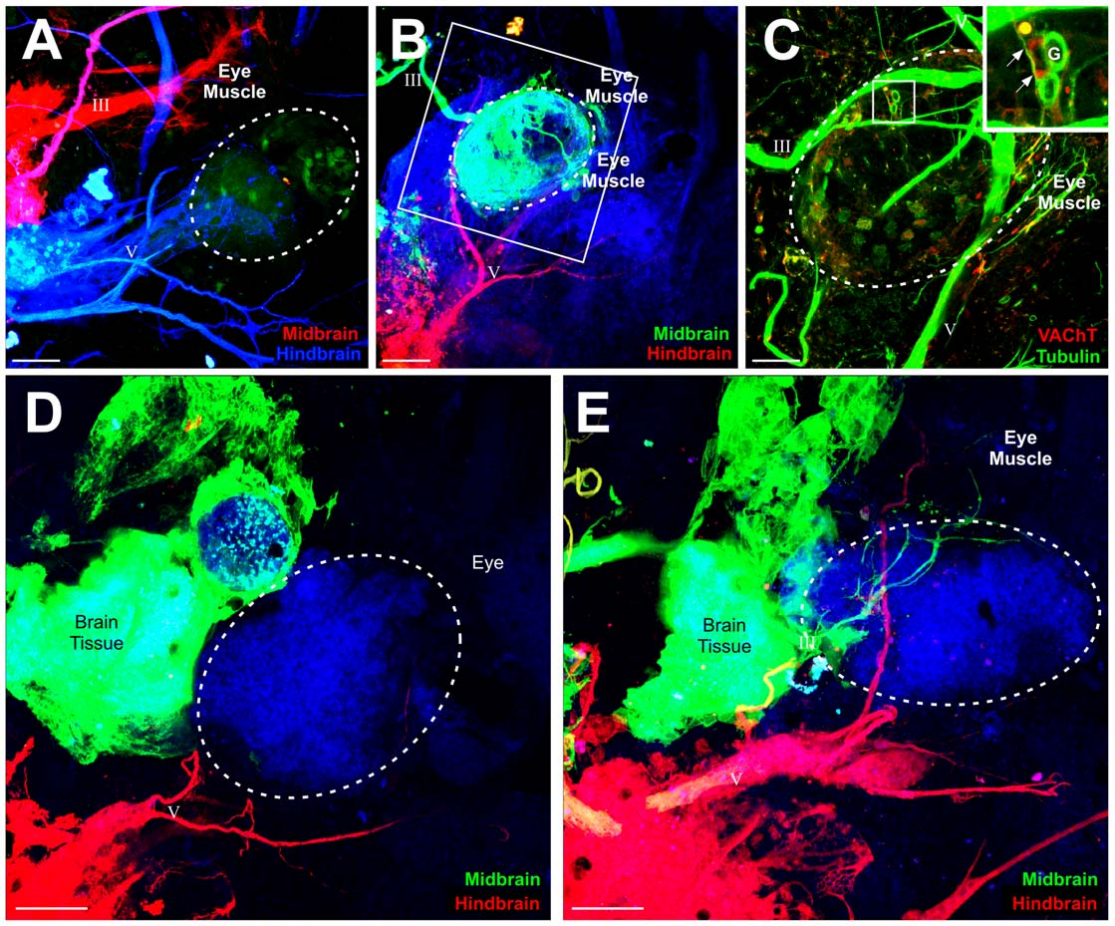
Transplanted tissue lacking nicotinic acetylcholine receptors such as heart and liver. **(A)** Implantations into the midbrain (red) and hindbrain (blue) revealed trigeminal innervation of the transplanted GFP-positive heart. The oculomotor (III) only innervated nearby eye muscle tissue. **(B)** Example of axons from the oculomotor nerve (III) projecting to a transplanted heart in addition to eye muscle. **(C)** Immunohistochemistry for tubulin (green) and VAChT (red) demonstrate that axons from the oculomotor nerve project to ganglion cells associating with the heart but not on the heart muscle itself. **(D)** Implantations into the midbrain (green) and hindbrain (red) revealed no axons projecting to the liver. **(E)** Implantations into the midbrain (green) and hindbrain (red) showed nerve fibers passing over the liver, but not innervating it. Transplanted tissue is circled. Scale bar is 50 µm in A, C; 100 µm in B, D, E.

Lipophilic dyes implanted into the midbrain and hindbrain revealed some axons projecting to transplanted heart tissue. Seven of 9 hearts had projections from the trigeminal nerve, as observed by dye implantations into the hindbrain (Fig. 5A). Five of the 9 hearts had projections from oculomotor neurons, as observed by implantations into the midbrain (Fig. 5B); however closer examination of these latter hearts showed that oculomotor neurons may be terminating on autonomic ganglia associated with the heart rather than on heart muscle itself (Fig. 5C). Thus, there were no clear examples of direct heart muscle innervation by motor neurons. Likewise, the heart beat was not changing when tadpoles moved around, suggesting limited effectiveness of oculomotor neurons to change the autonomous heartbeat frequency, when compensatory eye movements are initiated [32], [33].

Lipophilic dyes implanted into the hindbrain revealed very little innervation of the transplanted liver and no innervation of the liver was observed when lipophilic dyes were implanted into the midbrain. If there were apparent projections to the liver from the hindbrain, it was from a subset of trigeminal nerve axons. Trigeminal projections to the liver occurred in 11 of the 13 transplants. In 7 livers a subset of axons from the trigeminal just passed over the surface of the liver (Fig. 5E). The oculomotor nerve failed to innervate the transplanted liver in the 13 transplanted livers imaged and if it approached the liver, it would pass around or over to innervate the nearby eye muscles, if the latter were present (Fig. 5D and 5E).

## Discussion

The results here extend our previous work [24] by demonstrating that, not only spinal somatic motor neurons, but other subsets of motor neurons such as oculomotor motor neurons can also reroute and innervate the hair cells of the transplanted ear. In addition, we have tested for the ability of other targets and non-targets of motor neurons to receive novel innervation when similarly transplanted. Here we will discuss the range of innervation of novel tissues and its likely implications.

Using lipophilic dye implantations, we were able to demonstrate the ability of the oculomotor nerve, and in one case the trochlear nerve, to extend motor neuron axons to hair cells of an ear transplanted to the orbit to replace the eye. This finding, together with that of Elliott and Fritzsch [24], that spinal somatic motor neurons can innervate the ear when their normal target is no longer present, supports the idea that facial branchial motor neurons may have been rerouted to innervate the ear when the ear evolved in place of somites or somitomeres in ancestral vertebrates [11], [13], [34]. Together, these data demonstrate that spinal, branchial, oculomotor and thus likely any motor neuron have the ability to become an efferent to the ear; however, whether this is also true for visceral motor neurons projecting axons to neural crest derived ganglia [4], [12] remains to be seen. Unlike the ear, transplantation of somite, heart, and liver tissue into the orbit did not result in innervation by either the oculomotor or the trochlear nerve, with the exception of the autonomic parasympathetic ganglia associated with the heart which received axons from the oculomotor nerve in a few cases, possibly reflecting the parasympathetic component of the oculomotor nerve. Overall, this suggests that not all motor neurons can become efferents to any target, even if that tissue normally receives motor innervation, for example the somites. This is in line with previous reports of selective reinnervation of eye muscles after trochlear nerve transection which showed that in *Xenopus*, the trajectory of the nerve combined with the timing of innervation formation, determines the pattern of innervation by only one ocular nerve or several [35].

Unlike the facial branchial motor neurons which can innervate a variety of tissue types, including hair cells as efferents [11], the oculomotor and trochlear motor neurons normally innervate only specific eye muscles, with the exception of a parasympathetic branch of the oculomotor nerve that innervates the parasympathetic ciliary ganglion [36]. It is possible that this branch of the oculomotor was responsible for the innervation of parasympathetic ganglia associated with the heart tissue when transplanted to the orbit.

The inability of the oculomotor or trochlear axons to innervate somite-derived muscle tissue or of the spinal motor neurons to innervate branchial-arch-derived muscle tissue are likely due to differences in the origin of the tissues and may reflect an inability of cranial nerves to supply somite-derived tissue and of spinal motor neurons to supply branchial-arch-derived tissue. Such is the case with the trapezius muscle in normal development. The branchial-arch-derived trapezius muscle sits in the trajectory of spinal motor neurons, yet is innervated by motor neuron axons of the spinal accessory nerve or by a branch of the vagus [17], [18], [19]. Thus, it seems plausible that cranial motor neurons and spinal motor neurons rely on unique molecular signatures adapted by their target tissues to prevent cross-innervation at the head-neck boundary. What this signature is remains unclear. Data on experimental reorganization of ocular innervation in cases of loss of abducens or oculomotor innervation [23] suggests that some hierarchy of cross-innervation possibilities exist that need to be further investigated.

The ability of the hypoglossal motor axons to innervate somite-derived muscle tissue transplanted to the region previously occupied by the ear may reflect the natural ability of the hypoglossal motor neurons to innervate the somite-derived tongue muscle [37]. In aquatic organisms, such as *X. laevis*, the tongue is absent [38]. The hypoglossal nerve, without a target, may degenerate as nerve or motor neurons have not been found in adult *X. laevis* [39]; however, given the chance to innervate a target of similar origin (somite-derived muscle), the hypoglossal motor neurons apparently does so at least transiently. In a sense, motor neuron axons supplies of somite-derived muscle via the hypoglossal nerve, also recapitulates the original efferent innervation paradigm of the ear previously suggested which forced some facial motor neurons to innervate the ear in the absence of their likely somite derived original target [9].

As was observed in our previous work [24], afferents from transplanted ears can project to a novel area in the CNS. Although most afferents projected along the trigeminal nerve into the hindbrain as seen from both hindbrain and ear implantations with lipophilic dyes, some afferents from ears transplanted to the orbit projected back to the midbrain along the oculomotor or optic nerve. From there, the occasional axons projected into the forebrain. It appears that these afferents fasciculated with the nearest cranial nerve. That more axons followed the trigeminal nerve than any of the other nerves may be due to the larger territory of trigeminal projections than that of the others [40]. However, even with the same entry point into the brain along a given cranial nerve, there was no consistency once inside the brain for afferent axon projections.

The ability of the oculomotor and trochlear nerves to send projections to hair cells of the ear but not the other tissues directly suggests that there is something unique about the ear to allow for cross-innervation. One thing in common that all targets receiving direct motor input have is the presence of nicotinic acetylcholine receptors (nAChRs) [41], [42]. nAChRs are comprised of a pentamer of various subunits: α, β, δ, ε, and γ [43], [44], [45]. Of these subunits, the α subunit is required for the receptor to bind ACh [43]. The original α sequence has diversified, giving rise to the 10 different isoforms present today [43]. Of the 10 α subunits, α9 and α10 are the most diverged [46], [47], [48], [49]. Hair cells contain these most divergent of nAChR alpha subunits, α9 and α10 [46], [47], [48], [49]. It is possible that all motor neurons retain the ability to form synapses on α9 and α10-containg nAChRs. Mice in which the gene encoding the α9 nAChR subunit (*Chrna9*) or α10 nAChR subunit (*Chrna10*) was knocked out showed that these receptors were in part necessary for the development of synaptic connections between the olivocochlear efferents and the inner ear hair cells [47], [50], [51]. In mice lacking either α9 or α10 nAChR subunits, efferent synaptic contacts were larger in size but reduced in number compared to wild type littermates [50], [51]. No α9 or α10 double null mouse efferent innervation has been reported, leaving it open whether absence of both receptors eliminates all efferent synaptogenesis on hair cells. We are currently planning to knockdown both α9 and α10 nAChR subunits in *X. laevis* and transplant ears from these embryos into control embryos, either replacing the native ear or transplanting to the trunk to replace a somite or the orbit to replace the eye. The prediction would be that there is no efferent innervation of hair cells by any motor neurons, including an absence of hair cell innervation in the untransplanted ear. Future work would require also misexpressing α9 and α10 nAChR subunits in tissues that did not receive motor innervation when transplanted to either the orbit or trunk such to determine whether it is these nAChRs that allow for hair cell innervation by other motor neuron types.

In conclusion, the results presented here demonstrate the potential for a motor neuron to reroute to target a novel tissue that is transplanted into its trajectory; however, the nervous system is not completely plastic and not every motor neuron can interact with any target. Our data suggest that it is only the ear (and possibly parasympathetic ganglia) that can receive motor input from any motor neuron when placed in the trajectory of all motor neuron types tested here. The next step is to determine what properties present in the ear that are lacking in the other tissues transplanted that allow for synaptic formation by motor neurons. As previously mentioned, one strong candidate is the presence of the ancestral α9 and α10 nAChR subunits in the ear. Additionally there could be other unique components involved in synaptic formation as well as short range guidance cues to guide axons to the hair cells. Determining these factors may provide an additional understanding of the evolution of motor neuron innervation specificity of distinct peripheral targets. Furthermore, the insights generated here may be applicable in helping individuals with motor neuron damage regain function by rerouting other motor neurons to the denervated target tissue.
